# Role of fibrocytes and endothelial progenitor cells among low-differentiated CD34+ cells in the progression of lung sarcoidosis

**DOI:** 10.1186/s12890-020-01345-x

**Published:** 2020-11-20

**Authors:** Rutkowska Elżbieta, Kwiecień Iwona, Bednarek Joanna, Jahnz-Różyk Karina, Rzepecki Piotr

**Affiliations:** 1 Department of Internal Medicine and Hematology, Laboratory of Hematology and Flow Cytometry, Military Institute of Medicine, Warsaw, Poland; 2 Department of Internal Medicine, Pulmonology, Allergology and Clinical Immunology, Military Institute of Medicine, Warsaw, Poland; 3 Department of Internal Medicine and Hematology, Military Institute of Medicine, Warsaw, Poland

**Keywords:** CD34+ progenitor cells, Endothelial cells, Fibrocytes, Flow cytometry, Sarcoidosis

## Abstract

**Background:**

Sarcoidosis is a multisystemic granulomatous disease with still unknown etiology. Our previous studies showed a significantly higher percentage of CD34 + cells in the peripheral blood in patients with sarcoidosis (SA) compared to the control group. The objective of the present study was to characterized of the CD34 + cell population in peripheral blood in patients with SA with reference to the control group. Moreover in patients with SA, fibrocytes and endothelial cells were analysed and their relationship to the fibrosis process based on assessment of diffusing capacity for carbon monoxide (DLCO).

**Methods:**

Data from patients diagnosed with SA at Military Institute of Medicine (Warsaw, Poland) between January 2018 and December 2019 were collected and analysed ongoing basis. Peripheral blood was collected from 26 patients with newly diagnosed pulmonary SA and 16 healthy subjects. The immunomagnetic method and flow cytometry were used. Among the CD34+ progenitor cells were assessed: low-differentiated cells, hematopoietic progenitor cells and endothelial progenitor cells. The Statistica 12.0 software was used for a statistical analysis.

**Results:**

We observed a significantly higher percentage of low-differentiated cells (13.8 vs. 2.3, *P* = 0.001) and endothelial cells (0.3 vs. 0.0, *P* = 0.001) in patients with SA compared to the control group. In the study group the median proportion of fibrocytes was 1.877% (0.983–2.340) in patients with DLCO< 80%, while in patients with DLCO> 80% was 0.795% (0.139–1.951) (*P* = 0.72). The median proportion of endothelial progenitor cells was higher in patients with DLCO< 80%: 0.889% (0.391–1.741), than in patients with DLCO> 80%: 0.451% (0.177–0.857) (*P* = 0.44).

**Conclusions:**

In conclusion we demonstrated for the first time the immunophenotype of peripheral CD34 + cells with the degree of their differentiation. The study confirmed the involvement of low differentiated cells and endothelial cells in patients with SA.

## Background

Sarcoidosis is a systemic, multi-organ disease with granulomas formation. The most common localization in sarcoidosis (in 80–90% of patients) concerns the lungs and lymph nodes inside the chest. Sarcoidosis is diagnosed based on clinical, the histological evidence of noncaseating granulomas and radiological picture [1]. Granuloma in sarcoidosis consists of a core of epithelial histiocytes and multinucleated giant cells surrounded by CD4 + T cells and fibroblasts. Other cellular elements around the granuloma are CD8 + T cells, regulatory T cells, and B cells [2]. Interferon gamma and tumour necrosis factor- alfa are the major cytokines responsible for the granulomas formation [3]. There is a positive relationship between the extent of granuloma infiltration and the different inflammatory mediators [4]. Pulmonary sarcoidosis is characterized by increased T cells compared to healthy subjects in bronchoalveolar lavage fluid [5]. Some studies have shown lymphocytosis and elevated CD4/CD8 ratio in bronchoalveolar lavage fluid in patients which has been associated with a diagnosis of sarcoidosis. The pathogenesis of sarcoidosis involves the activation of T lymphocytes and macrophages which release pro-inflammatory cytokines, enzymes and chemokines that modulate inflammation and lipid metabolism [6]. Changes in lipid metabolism may be also important in sarcoidosis pathogenesis. It is known that alterations of lipid metabolism are associated with damage of the plasma membrane, lung and bronchial capillary endothelial cells [7]. During the last years the new diagnostic methods have been discovered, but markers in peripheral blood (PB) of the sarcoidosis are still under studied. The role of individual populations in the development of inflammatory changes in sarcoidosis is unclear. Our previous studies showed a significantly higher percentage of CD34 + progenitor cells in the PB in patients with newly diagnosed sarcoidosis compared to the control group and showed a positive correlation with CD4/CD8 ratios. CD34+ progenitor cells may be important in the pathogenesis of sarcoidosis [8]. The PB cell population expressing the CD34+ antigen is a heterogeneous group, which may include both the least differentiated pluripotent stem cells and cells acquiring markers of specific cell lines: myeloid progenitor cells and lymphoid progenitor cells. In physiological conditions the CD34+ antigen is present on the surface of young hematopoietic cells, endothelial cells: circulating (CECs) and progenitor endothelial cells (EPCs) and fibrocytes in PB [9, 10]. Studies from recent years provide new data on the involvement of cells with bone marrow origin in interstitial diseases with pulmonary fibrosis [11–13]. Granulomatous inflammation can develop into pulmonary fibrosis - this occurs in about 20% of patients with sarcoidosis [14, 15]. In the overwhelming majority of cases, sarcoidosis affects the chest organs - including the lungs. Sarcoidosis can occur with pulmonary fibrosis, which is the result of chronic inflammation. The fibrocytes leaving the bone marrow become circulating mesenchymal progenitor cells associated with several fibrotic disorders, such as pulmonary fibrosis. They are the precursors of fibroblasts that produce tissue proteins in the form of collagen. These cells have been detected in the blood by flow cytometry using panel of monoclonal antibodies: CD34+, CD45+, collagen-1+. Circulating fibrocytes, are thought to be able to differentiate into fibroblasts and cause collagen accumulation in injured organs [16]. Recent data obtained from studies assessing the role of fibrocytes in diseases occurring with fibrosis of the lung parenchyma suggest that there is a relationship between the severity of the fibrosis process and the presence of increased number of fibrocytes in PB [17]. No literature characterizing the population of CD34+ cells in patients with sarcoidosis has been found.

Moreover fibrogenesis is a mechanism of wound healing, which is associated with vascular remodelling. CEC cells may be biomarkers of the disease with fibrosis and show the balance between vascular damage and repair [18, 19] while EPCs appear to be essential in maintaining vascular homeostasis. EPCs are released from bone marrow and lung resident structures, but their role in chronic lung disease is controversial [20, 21]. The amount of EPCs may vary in chronic obstructive pulmonary disease [22] as well as in patients with hypoxemia and severe restrictive lung disease [23], but little is known about the role and amount of EPCs in sarcoidosis.

Despite the enormous progress of research in sarcoidosis, pathogenesis has not been established. We also do not have non-invasive markers to monitor the disease or predict its progression and irreversible pulmonary fibrosis.

The aim of the present study was to evaluate the immunophenotype of the CD34+ cell population in peripheral blood in patients with newly diagnosed sarcoidosis with reference to the control group. Moreover in patients with diagnosed lung sarcoidosis, fibrocytes and EPCs were analysed and their relationship to the fibrosis process based on assessment of diffusion lung capacity for carbon monoxide (DLCO).

## Methods

### Patients

The study group consisted of 26 patients with newly diagnosed pulmonary sarcoidosis. There were 15 women and 11 men; mean age: 45.2 ± 12.2 years; range (min-max): 29–70 years. There were patients in stages I-III of the disease: 17 patients in stage II, 7 in stage I and 2 patients in stage III, hospitalized in Department of Internal Medicine, Pulmonology, Allergology and Clinical Immunology, Military Institute of Medicine (from January 2018 to December 2019). Each patient had provided written informed consent (the Military Institute of Medicine Ethics Committee nr 37/WIM/2015) before each diagnostic procedure. The control group included 16 healthy subjects (10 women, 6 men), mean age: 40.5 ± 11.5 years; range (min-max): 29–60 years.

Primary sarcoidosis were confirmed by histopathology. DLCO were measured to each patients according to American Thoracic Society/European Respiratory Society guidelines [24]. DLCO evaluates the alveolar-capillary integrity and reflects the surface area and pulmonary capillary blood volume available for gas exchange [25]. Patients with pulmonary sarcoidosis were divided into two groups depending on the DLCO value, I group: DLCO> 80% and II group: DLCO< 80%.

Tests were performed in peripheral blood collected on sodium heparin Vacutainer CPT Ficoll tubes BD (Becton Dickinson, Franklin Lakes, New Jersey, United States).

Patient and Public Involvement: No patient involved.

### Immunomagnetic separation

**Fig. 1:**
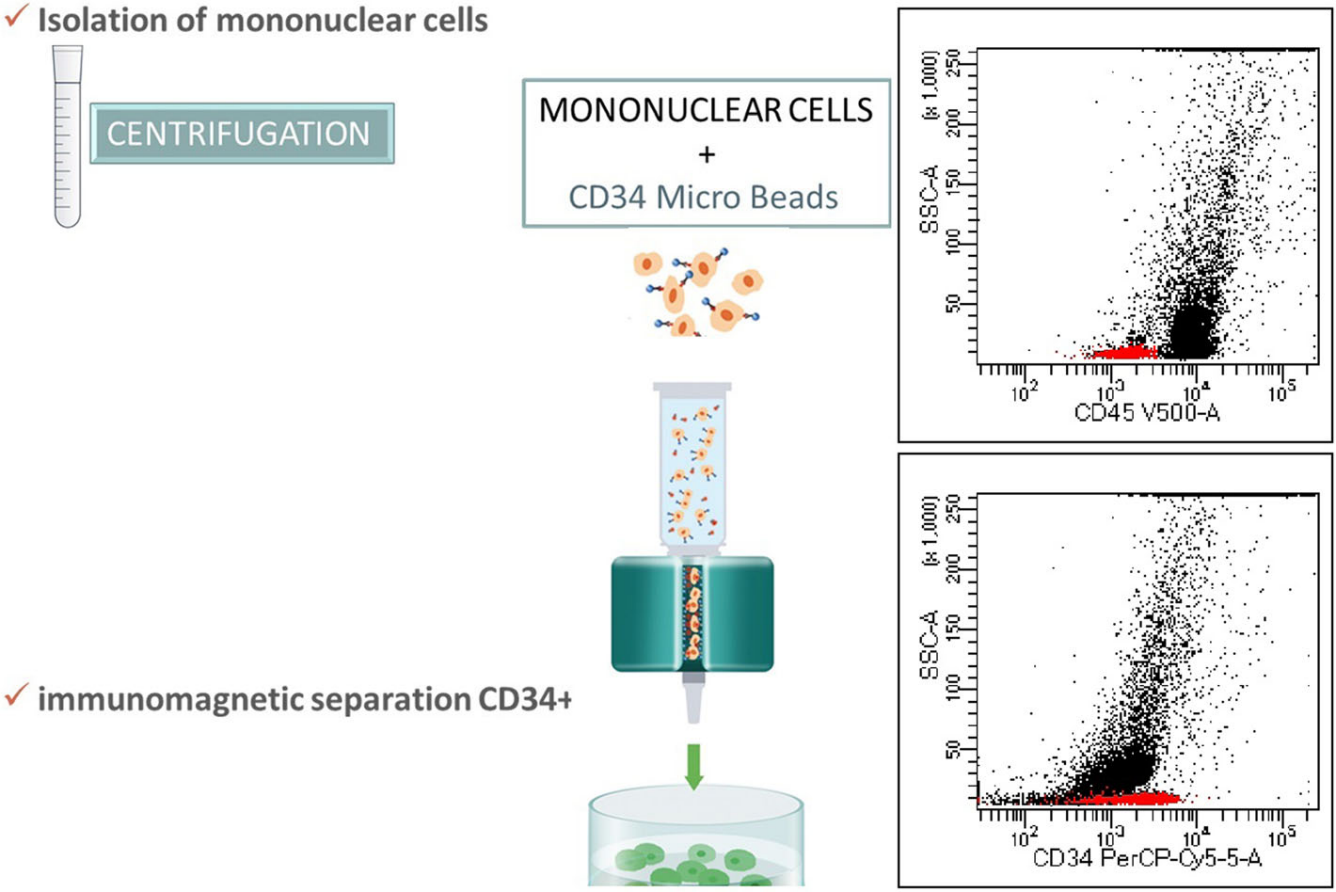
Schematic determination of CD34+ cells using the immunomagnetic separation (IMS) and flow cytometry (FC)

Due to the exceptional rarity of CD34+ progenitor cells in the peripheral blood, a precise isolation method was used - positive selection of CD34+ cells by the immunomagnetic method (IMS) (Direct CD34 Progenitor Cell Isolation Kit, Miltenyi Biotec, Bergisch Bladbach, Germany). To achieve the required efficiency of this process, initial separation of mononuclears from peripheral blood by management gradient centrifugation was used. In the next step cells were washed once by phosphate-buffered saline and resuspended in a final volume of 300 μL of buffer up to 10^8^ cells. FcR Blocking Reagent and CD34 Micro Bead were added and incubated for 30 min in the 2–8 °C. Column in the magnetic field was placed on suitable MACS Separator, rinsing with appropriate amount of buffer and cell suspension was applied on the column. After washing, unlabelled cells were passed through and magnetically labelled cells were flashed out, washed and ready for staining with antibodies (Fig. 1).

The material prepared in this way was subjected to multi-parameter analysis using 8-color flow cytometry (FC).

Flow cytometry.

CD34+ progenitor cells surface and intracellular antigens were determined by FC using FACS Canto II BD flow cytometer (Becton Dickinson, Franklin Lakes, New Jersey, United States). The FC with a panel of monoclonal antibodies were used for CD34+ cells identification. For surface markers detection, cells were stained with fluorescently labeled antibodies: CD45-V500, CD34- PerCP-Cy5.5, CD133-PE, CD38-APC-H7, HLA-DR-V450, CD117-PE-Cy7, CD33-APC, CD22-PE, CD19- FITC, CD309-APC (BD Biosciences) for 20 min at room temperature. After washing, cells were analyzed within 2 h. For intracellular markers detection: collagen-1-FITC (Sigma-Aldrich, St. Louis, MO, Unite States) the additional step with IntraStain (Dako, Glostrup, Denmark) for fixation and membrane permeabilization was done.

For each sample, a minimum of 100,000 events were collected. Data were analysed with DIVA Analysis software 8.0.1 (Becton Dickinson) and Infinicyt 1.8 Flow Cytometry (Cytognos, Salamanca, Spain). Internal quality control was performed daily by checking the cytometer’s optical detector and aligning lasers and fluid systems using CS&T IVD Beads BD FACS Diva (Becton Dickinson), respectively, according to manufacturer guidelines.

Among the CD34+ progenitor cells were assessed:

- low-differentiated cells: CD45 + dim CD133+ CD34+ HLA-DR+ CD38-,
- myeloid progenitor cells: CD45 + dim CD34+ CD133+/− CD38+/− HLA-DR+ CD117+ CD33−/+dim,
- lymphoid progenitor cells: CD45 + dim CD34+ CD133+/− CD38+/− HLA-DR+ CD22 + dim CD19-
- endothelial cells: CD45 + dim CD34+ CD309+

Moreover among the CD34+ low-differentiated cells were assessed:

- EPC: CD45 + dim CD34+ CD133+ CD38- CD309+
- fibrocytes: CD45 + dim CD34+ CD133+ CD38- collagen-1+ CD309-

### Statistical analysis

The Statistica 12.0 software (TIBCO Software, Palo Alto, California, Unite State) was used for a statistical analysis. The results are expressed as medians with interquartile range (Q1-Q3) for continuous variables Comparisons between groups regarding continuous variables were made using the Mann-Whitney test. Relations between the quantitative variables were analyzed by Spearman correlations. Significance was assumed when a null hypothesis could be rejected at a *P* value of less than 0.05.

## Results

The characteristics of the investigated group were summarized in Table 1. The women were more numerous compared to the men (*P = 0.04*). Most patients were in II stage of sarcoidosis. Due to a small number of patients in each group we did not perform a comparison between groups with different stages of the disease.

**Table 1:** Characteristic of the study population with sarcoidosis

	Patients
Sex: F/M (n)	15/11
Age (mean ± SD years)	45.2 ± 12.2
Women (mean ± SD years)	42.6 ± 11.8
Men (mean ± SD years)	46.7 ± 12.9
DLCO (%) (mean ± SD)	87.5 ± 17.1
- > 80% (n, mean ± SD)	- 15, 96.8 ± 6.3
- < 80% (n, mean ± SD)	- 11, 64.2 ± 11.7
Clinical symptoms (%)	
- Cough	15.8%
- Dyspnoea	26.3%
- Lymphadenopathy	100.0%
- Pulmonary fibrosis	9.3%
Stage of disease (n,%)	
I	7 (26.9%)
II	17 (65.4%)
III	2 (7.7%)

Abbreviations: *F* female, *M* male, *DLCO* Diffusing capacity for carbon monoxide

**Fig. 2:**
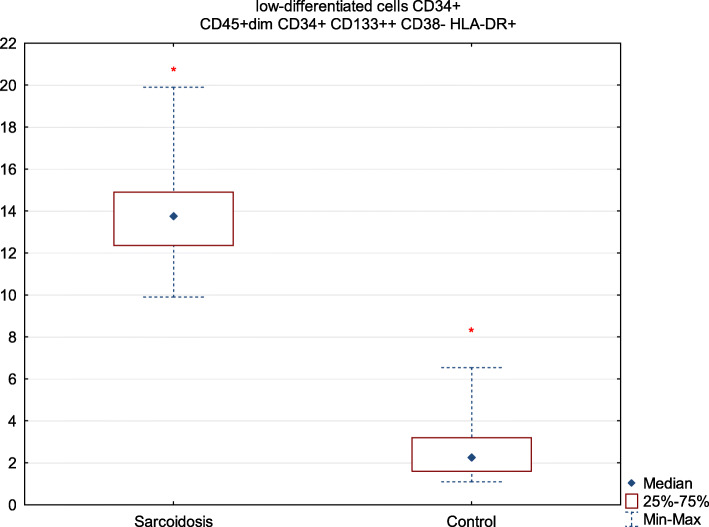
Median proportion of low-differentiated cells: CD45 + dim CD133+ CD34+ HLA-DR+ CD38- in patients with sarcoidosis and in control groups. Data are presented as median values (Q1-Q3) (minimum-maximum) (* *p* < 0.05)

We observed a significantly higher percentage of low-differentiated cells (13.8 vs. 2.3, *P* = 0.001) in patients with sarcoidosis compared to the control group (Fig. 2).

**Fig. 3:**
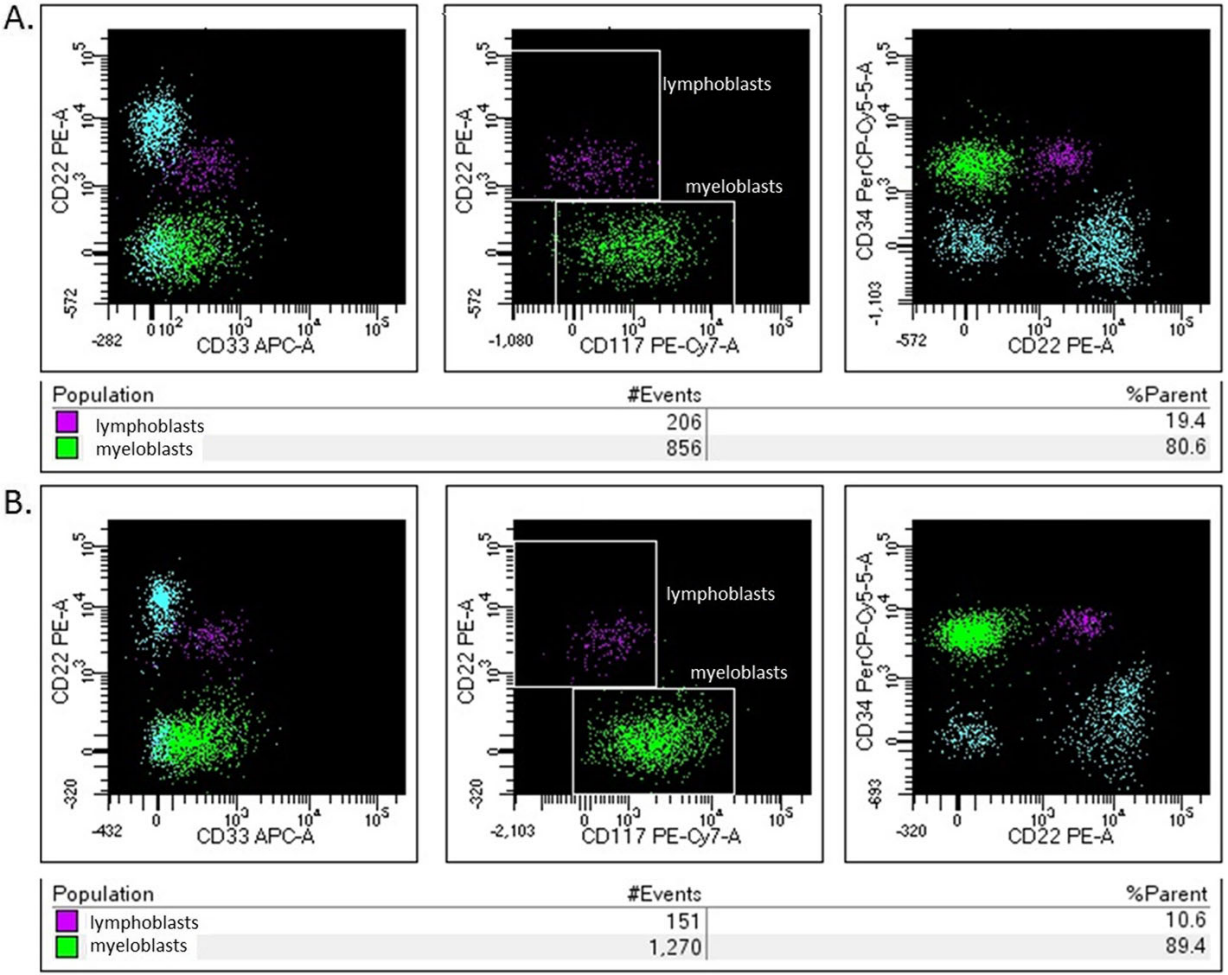
The myeloid progenitor cells and the lymphoid progenitor cells in one heathy donor – **a**., and patient with sarcoidosis – **b**. (example of histogram from flow cytometry analysis)

Among the hematopoietic cells, the population of the myeloid line was predominated in both groups: patients and control group (Fig. 3). There were 83.3% (79.7–84.3) myeloid progenitor cells in study group and 88.2% (81.9–91.4) in control group (*P* = 0.37). The lymphoid progenitor cells were 16.8% (15.7–20.3) in patients with sarcoidosis and 12.5% (8.8–18.1) in healthy donors (*P* = 0.37).

We noticed a higher proportion of endothelial cells in patients with sarcoidosis compared to the control group (0.3 vs. 0.0, *P* = 0.001).

The average proportion DLCO for the all study group was 87.5 ± 17.1. In the next stage of the study patients with pulmonary sarcoidosis were divided into two groups depending on the DLCO value. There were two groups: I- patients with DLCO> 80% (*n* = 15) and II- patients with DLCO< 80% (*n* = 11). Fibrocytes and EPC were analyzed among low-differentiated CD34+ cells in these two groups with sarcoidosis.

**Fig. 4:**
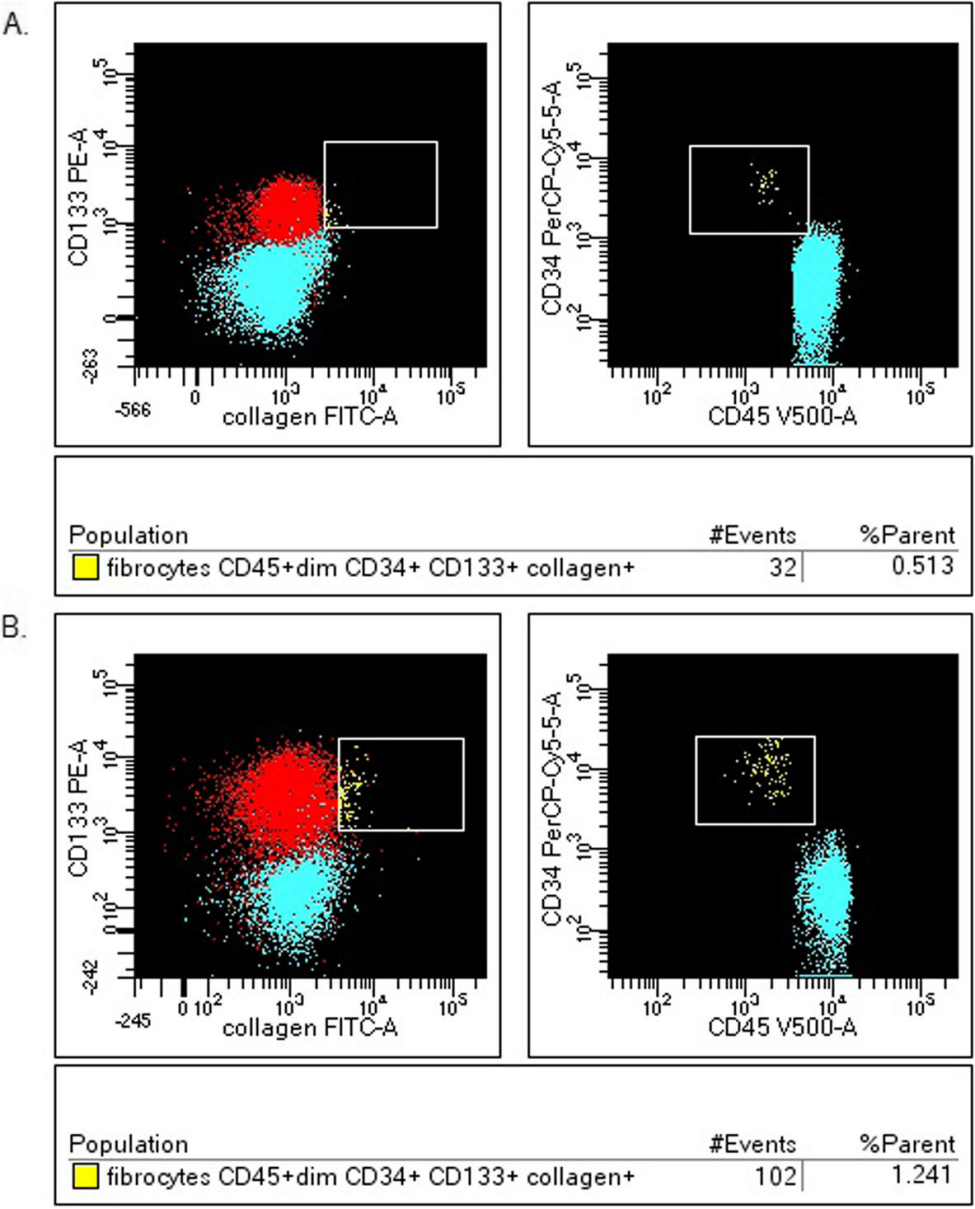
The fibrocytes in the one patient with DLCO> 80% – **a**., compared to the one patient with DLCO< 80% – **b**. (example of histogram from flow cytometry analysis)

**Fig. 5:**
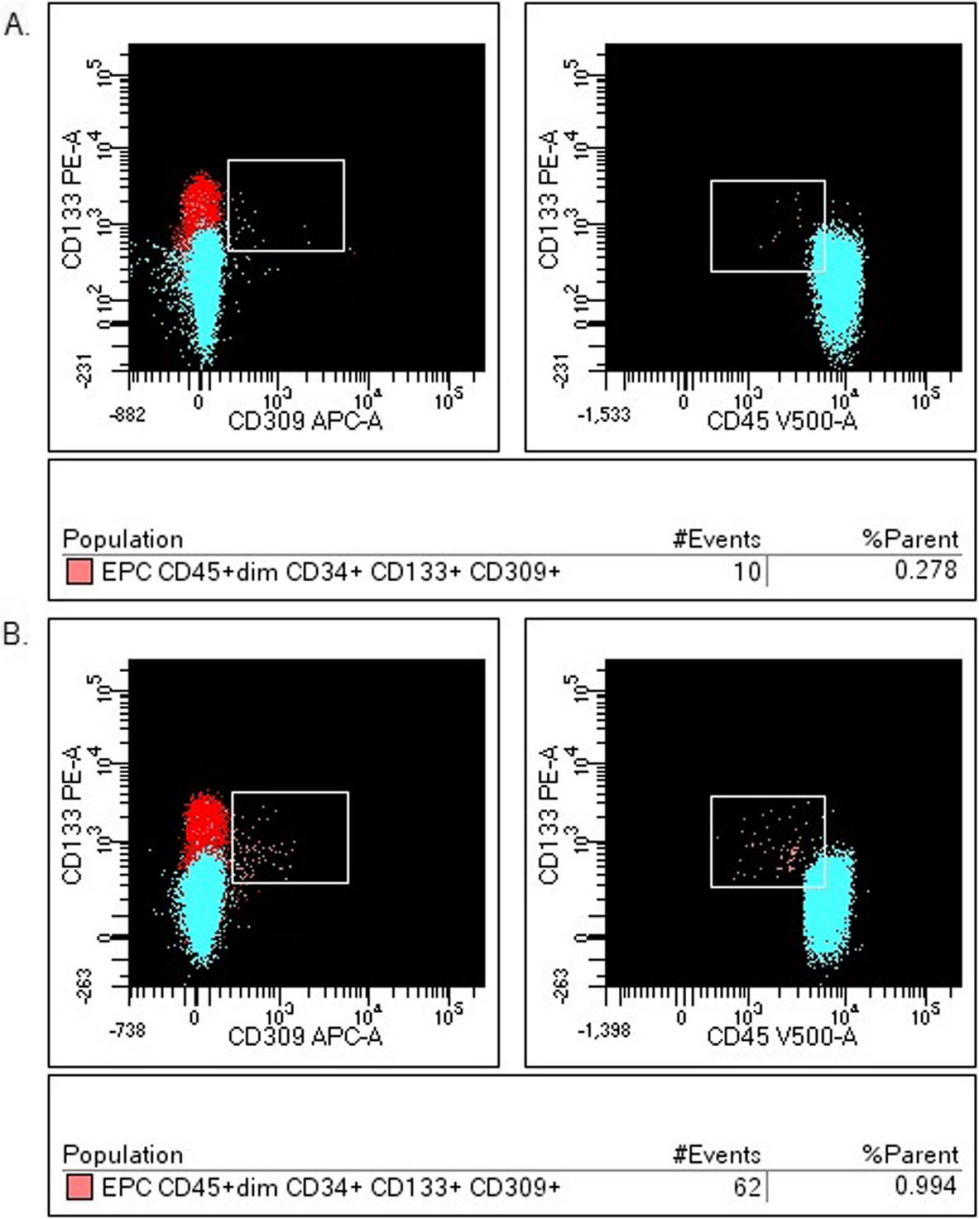
The endothelial progenitor cells (EPC) in the one patient with DLCO> 80% – **a**., compared to the one patient with DLCO< 80% – **b** (example of histogram from flow cytometry analysis)

Among all low-differentiated cells CD34+, the median proportion of fibrocytes was 1.309% (0.288–2.048) and EPC accounted for 0.519% (0.231–1.317) in all patients (group I and II) with sarcoidosis. The median proportion of fibrocytes was 1.877% (0.983–2.340) in patients with DLCO < 80%, while in patients with DLCO> 80% was 0.795% (0.139–1.951) (*P* = 0.72) (Fig. 4). The median proportion of EPC was higher in patients with DLCO< 80%: 0.889% (0.391–1.741), than in patients with DLCO> 80%: 0.451% (0.177–0.857) (*P* = 0.44) (Fig. 5).

Moreover we observed a significant negative correlation between the proportion of EPC and DLCO in patients with sarcoidosis (*r* = − 0.4, *P = 0.03*).

## Discussion

This study was to evaluate the immunophenotype of the CD34+ cell population in PB in patients with newly diagnosed sarcoidosis with reference to the control group and exact characteristics low-differentiated CD34+ cells including DLCO values in sarcoidosis.

The study group consisted of patients with confirmed pulmonary sarcoidosis in mainly II stages of the disease. The ratio of women to men was 15:11 which corresponds with literature data. Sarcoidosis is more common in women, particularly in patients who present after age of 50 years [26]. Pulmonary sarcoidosis often has an insidious onset with more common symptoms. Our patients report some non-significant symptoms of respiratory system. In study group confirmed the presence of symptoms such as: cough in only about 16% patients and dyspnea in about 26% patients. In literature typical pulmonary symptoms in sarcoidosis patients are not typic, include nonproductive cough, chest discomfort, exertional dyspnea and wheezing [1]. Pulmonary fibrosis was observed in only 1 patients from the study group.

The present study for the first time showed higher proportion of low-differentiated cells (13.8 vs. 2.3, *P* = 0.001) in patients with sarcoidosis compared to the control group. The heterogeneous CD34+ cells population was divided and characterized in PB patients with sarcoidosis. Previous studies have shown that in untreated patients with newly diagnosed pulmonary sarcoidosis, the number of CD34+ peripheral blood cells is significantly higher compared to the control group [8].

In this study we used for the first time IMS for CD34+ cells identification. The set of both methods: IMS and FC, allowed for quick and effective enrichment of the accurately characterized CD34+ progenitor cells in PB.

There was a greater proportion of myeloid progenitor cells than the lymphoid progenitor cells, both in the study and control group. These results implied that the dominant population among CD34+ cells was myeloid progenitor cells, regardless of the clinical condition and presence of the disease. This observation showed that specific targeted hematopoietic CD34+ cells: myeloid progenitor cells and lymphoid progenitor cells are not as sensitive to the disease environment as low-differentiated CD34+ cells. Similar data have not been found in sarcoidosis in the available literature.

Next steps in this study we used DLCO values to divide and accurately characterize patients with sarcoidosis. The measurement of DLCO (transfer factor for carbon monoxide or TLCO), is used to assess the vesicular-capillary barrier function that separates air in the alveoli from blood in the alveolar capillaries. DLCO gives important information about the size and integrity of the alveolar blood membrane. DLCO has a high value in the diagnosis of interstitial lung diseases such as sarcoidosis, idiopathic pulmonary fibrosis, allergic alveolitis and others such as anemia and congestive heart failure [25]. The majority of sarcoidosis patients have normal pulmonary function testing, but may also show a restrictive or obstructive pattern. Reduction in DLCO is observed in patients with sarcoidosis and low DLCO score is associated with severity of the disease in sarcoidosis patients. This is one of the most common abnormalities that can result from parenchymal involvement or lead to pulmonary hypertension [27, 28].

We used the recommend algorithm to interpret DLCO %. Patients with DLCO ≥80% were normal and patients with DLCO < 80% was reduced diffusion [29]. We did not classify patients with DLCO < 80% into mild, modern, and severely reduced diffusion considering the small number of patients in both groups. In this study we observed reduced DLCO values in about 40% patients (64.2 ± 11.7). The average proportion DLCO for the all study group was 87.5 ± 17.1.

Other authors presented that DLCO is reduced in at exercise in patients with sarcoidosis and is highly predictive of gas exchange abnormalities. Together with desaturation at exercise is a strong functional parameter and may correlate with the severity and extent of sarcoidosis [30].

In this study, among low-differentiated cells CD34+ the median proportion of fibrocytes was 1.309% (0.288–2.048) and the median proportion of EPC was 0.519% (0.231–1.317). We observed differences between patients considering the DLCO value. Patients with reduced diffusion (DLCO< 80%) had higher proportion of fibrocytes and EPC cells then patients with DLCO> 80%. An increased proportion of EPC and fibrocytes in patients with reduced diffusion may indicate the role of these cells in sarcoidosis pathogenesis and be used as markers for assessing the severity of the disease. In the recent years obtained results of clinical studies gave us many reasons to believe that there is a relationship between the present of CD34+ fibrocytes and EPCs in PB and degree of severity of pulmonary fibrosis, especially in patients with idiopathic pulmonary fibrosis (IPF). We did not find any reference to this observation in sarcoidosis. Mehrad et al. [31] demonstrated for the first time that fibrocytes play a key role in both physiological and abnormal fibrosis. They focused on the role of fibrocytes in the lungs fibrotic diseases, such as: interstitial lung diseases, pulmonary hypertension, asthma without observation in sarcoidosis. Another author showed that the number of fibrocytes increases in the lungs and attracted to the lungs in response to CXCL12 and are involved in fibrosis in IPF [32]. Heukels P et al. [17] also have shown that percentage of lung fibrocytes was 2,6% of all CD45+ cells in IPF lungs, which was statistically increased compared with control lungs. They suggested that fibrocytes could be used as a possible diagnostic marker and target for therapy. In fibrotic lung disease, an increased number of fibrocytes can lead to pulmonary fibrosis. Moreover, other authors have shown that increased levels of peripheral blood fibrocytes in IPF patients are associated with poor prognosis [33]. Fibrocytes are increased in acute phase of the IPF, but there was no changes in fibrocyte numbers in patients with acute respiratory distress syndrome [34].

The next observation in this study was higher proportion of EPC cells in patients with reduced diffusion compared to normal diffusion. Moreover we observed a significant negative correlation between the proportion of EPCs and DLCO values in patients with sarcoidosis.

Smadja MD et al. also have shown that in patients with a low diffusing of DLCO< 40%, EPCs were higher compared to those with DLCO> 40%. EPCs were negatively correlated with DLCO, but this study group had idiopathic pulmonary fibrosis. We did not find in the literature studies of EPCs in patients with sarcoidosis. This is innovative in our study. This relationship between the presence of EPCs in PB and DLCO value may be related to the severity of the disease, but no data on whether elevated EPC cells level is a positive or negative factor in sarcoidosis was found.

The endothelium plays a key role in the regulation of pulmonary vessels, and endothelial dysfunction is seen as crucial to the initiation and progression of lung diseases, especially in IPF. In other studies was shown that CECs closely correlated with the state of activation of the pulmonary endothelium [35, 36].

Despite the differences in characterizing and defining CECs, some data suggests that these cells play an important role in diseases, such ascardiovascular and cerebrovascular, endocrinological, haematological and connective tissue disorders [37]. The number of EPCs is reduced in patients with chronic heart failure [38] stroke [39] insulin-dependent and non-insulin-dependent diabetes [40, 41] rheumatoid arthritis [42] and chronic renal failure [43]. On the other hands, as recently reported, EPCs are increased in patients with acute myocardial infarction and unstable angina. Most reports described EPC as a positive factor. The above works suggest that under physiological conditions, EPCs play a major role in vascular integrity [44–46].

Potential uses of bone marrow derived EPCs have been demonstrated in other studies.

Important for therapeutic success is the evaluation and understanding of EPC homing, differentiation and activation, which remains unclear, especially in sarcoidosis.

Our results and the above data suggest that fibrocytes and EPCs are strongly related to the DLCO values in patients with sarcoidosis, but more research is needed to understand the pathophysiology of sarcoidosis, particularly in relation to systemic disease involvement.

The limitation of this study was a small number of patients and a lack of possibility to subgrouping them according to stage of the disease. However, the results were the first and set the direction for further research.

## Conclusion

This study demonstrated for the first time the characterization of immunophenotype of peripheral CD34+ cells with the degree of their differentiation in patients with sarcoidosis. The above results indicated the possible involvement of low- differentiated cells in patients with sarcoidosis mainly endothelial cells and fibrocytes in patients with sarcoidosis. These cells may be an early parameter indicating a negative progression of the disease.

This observation needs further studies of usefulness of these cells in the diagnosis, monitoring and prognosis of patients with sarcoidosis.

## Data Availability

The datasets used and/or analysed during the current study are available from the corresponding author on reasonable request.

## Abbreviations

ARDS: Acute respiratory distress syndrome
ATS: American Thoracic Society
BALF: Bronchoalveolar lavage fluid
CEC: Circulating endothelial cells
DLCO: Diffusing capacity for carbon monoxide
EPC: Endothelial progenitor cells
ERS: European Respiratory Society
FC: Flow cytometry
IMS: Immunomagnetic separation
INF-γ: Interferon gamma
PB: Peripheral blood
